# Comparison of Stress and Suicide-Related Behaviors Among Korean Youths Before and During the COVID-19 Pandemic

**DOI:** 10.1001/jamanetworkopen.2021.36137

**Published:** 2021-12-13

**Authors:** So Young Kim, Hye-Rim Kim, Bumjung Park, Hyo Geun Choi

**Affiliations:** 1 Department of Otorhinolaryngology–Head & Neck Surgery, CHA Bundang Medical Center, CHA University, Seongnam, Korea; 2 Department of Pediatrics, CHA Bundang Medical Center, CHA University, Seongnam, Korea; 3 Department of Otorhinolaryngology–Head & Neck Surgery, Hallym University Sacred Heart Hospital, Anyang, Korea; 4 Hallym Data Science Laboratory, Hallym University College of Medicine, Anyang, Korea

## Abstract

**Importance:**

The COVID-19 pandemic has had a substantial psychological effect on young people. A quantitative assessment of the association between the pandemic and stress and suicidality in youths is needed.

**Objective:**

To investigate the association of the COVID-19 pandemic with self-reported stress and suicide-related behaviors in youths.

**Design, Setting, and Participants:**

This cross-sectional study used data from the the Korea Youth Risk Behavior Web-based Survey (KYRBWS) conducted in 2019 and 2020 with youths aged 12 to 18 years. Statistical analysis was performed from January to February 2021.

**Main Outcomes and Measures:**

The odds ratios (ORs) with 95% CIs for subjective stress level, sadness or despair, suicidal thoughts, suicide planning, and suicide attempts among 2020 participants were compared with those of the 2019 participants using multiple logistic regression analysis with complex sampling using weighted values.

**Results:**

The 48 443 youths in the 2019 KYRBWS (24 917 male youths [51.3%]; mean [SD] age, 15.0 [1.7] years) and the 44 216 youths in the 2020 KYRBWS (23 103 male youths [52.5%]; mean [SD] age, 15.1 [1.7] years) were compared. The degree of subjective stress was lower in the 2020 participants than in the 2019 participants (severe stress: adjusted OR [aOR], 0.90 [95% CI, 0.83-0.97]; very severe stress: aOR, 0.65 [95% CI, 0.60-0.72]). Sadness or despair was also lower in the 2020 participants than in the 2019 participants (aOR, 0.81 [95% CI, 0.78-0.84]). There were fewer suicide-related behaviors, including suicidal thoughts, suicide planning, and suicide attempts, among the 2020 participants than among the 2019 participants (suicidal thoughts: aOR, 0.77 [95% CI, 0.73-0.80]; suicide planning: aOR = 0.88 [95% CI, 0.81-0.96]; suicide attempts: aOR, 0.64 [95% CI, 0.58-0.70]).

**Conclusions and Relevance:**

This cross-sectional study found that severe stress, sadness or despair and suicide-related behaviors had inverse associations with the early COVID-19 pandemic in Korean youths. These findings suggest that levels of high stress decreased among Korean youths in the early period of the pandemic compared with prepandemic levels.

## Key Points

**Question:**

Was the COVID-19 pandemic associated with suicidality the COVID-19 pandemic in youths?

**Findings:**

In this cross-sectional study of 92 659 Korean youths, suicidality was lower during the early COVID-19 pandemic period compared with the prepandemic period. Thoughts of suicide, plans of suicide, and suicide attempts among 2020 participants were less common compared with 2019 participants.

**Meaning:**

These findings suggest that high levels of stress decreased during the early COVID-19 pandemic period compared with prepandemic stress levels.

## Teaser

This cross-sectional study examines the association of the early COVID-19 pandemic period and self-reported stress and suicide-related behaviors among Korean youths aged 12 to 18 years.

## Introduction

The COVID-19 pandemic has affected every aspect of human life, including social activity, education, and psychological problems.^1,2^ The impacts of the COVID-19 pandemic on these factors have been observed to be different according to personal characteristics and social circumstances.^3^ In US adults, individuals with more physical exercise, higher familial support, and better quality of sleep were found to have greater psychological resilience during the lockdown period of the COVID-19 pandemic.^3^ The increased psychological burden during the COVID-19 pandemic was shown to increase the risk of suicidality.^4,5,6^ A number of recent studies reported an increase in the suicide rate during the COVID-19 pandemic across all age populations^4^ and adult populations.^5,7^ However, the impact of the COVID-19 pandemic on suicidality may differ according to socioeconomic factors. Suicidality was found to be unchanged or decreased in high- to upper-middle-income countries during the early phase of the COVID-19 pandemic.^8^

Suicide has been reported as the second most common cause of death among adolescents in the US.^9^ In Korea, suicide has been reported to be the leading cause of death among adolescents.^10,11^ The risk factors for suicide attempts are multifactorial and include individual psychologic factors, drug abuse, and sleep problems.^12,13^ Therefore, both personal and socioenvironmental factors, such as the occurrence of the COVID-19 pandemic, may have an effect on suicide attempts in adolescents. Several studies have estimated the effects of the COVID-19 pandemic on suicidality in adolescents.^12,14^ Increased anxiety and distress, economic adversity, and limited social support have been identified as risk factors for suicidality in adolescents during the COVID-19 pandemic.^14^ In youths who are psychologically vulnerable, suicidal ideation and suicide attempts were found to be more common during the COVID-19 pandemic than in matched adolescents during the pre-COVID-19 era.^15^ On the other hand, fewer academic and social pressures due to school lockdown have been predicted to be protective factors for suicidality in adolescents during the COVID-19 pandemic.^14,16^ Among children and adolescents in Japan, there was no significant change in the suicide rate during the COVID-19 pandemic (March to May 2020).^12^

We postulated that there may be mixed impacts of the COVID-19 pandemic on stress and suicidality in youths. The restriction of physical and social activities due to the COVID-19 pandemic and increased concerns about SARS-CoV-2 infection could escalate stress and suicidality in youths. On the other hand, school lockdowns and increased leisure time could reduce stress and suicidality. To test the association between the COVID-19 pandemic and stress and suicidality in Korean youths aged 12 to 18 years, we compared their stress levels and suicidality before and after the COVID-19 outbreak. In Korea, the first patient with COVID-19 was diagnosed on February 19, 2020. Thus, this study compared stress levels and suicidality between 2019 and 2020 participants. Because sex differences in stress responses have been reported, subgroup analyses were conducted according to sex. In addition, the impacts of COVID-19 could differ according to income level; thus, different income levels were considered for subgroup analyses. Moreover, because the stress from academic burden could differ according to school performance, the association between stress and suicidality and the COVID-19 pandemic was also analyzed according to the level of school performance.

## Methods

### Study Population and Data Collection

The ethics committee of Hallym University approved the use of these data. The study was exempted from the need for written informed consent by the Hallym University institutional review board. The Korea Youth Risk Behavior Web-based Survey (KYRBWS) data from 2019 and 2020 were used (eAppendix in the Supplement).^17^ The KYRBWS is a school-based, nationwide, web survey in youth. Annually, nationally representative youth were sampled, with participation rates as high as 95% to 99% in Korea. An anonymous self-report survey was conducted to assess health-risk behaviors, including smoking, alcohol consumption, and psychological behaviors. This study followed the Strengthening the Reporting of Observational Studies in Epidemiology (STROBE) reporting guideline for cross-sectional studies.

Of the 112 251 total participants (57 303 in 2019; 54 948 in 2020), some participants were excluded from this study owing to the following criteria: a lack of information on age (n = 373), height or weight (n = 2596), sedentary time (n = 3682), and sleep time (n = 12 941). Finally, 92 659 participants (48 443 in 2019; 44 216 in 2020) aged 12 to 18 years old were included in this study (Figure). We analyzed the prevalence of subjective stress; sadness or despair; and thoughts, planning, and suicide attempts between 2019 and 2020.

**Figure.**
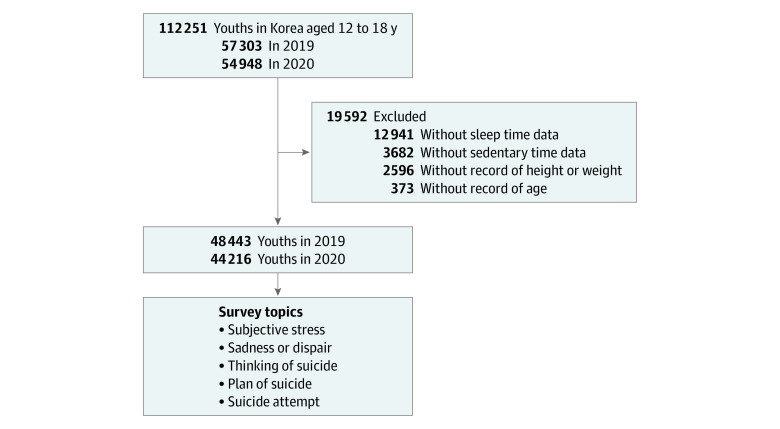
Study Population Korean youths aged 12 to 18 years in the Korea Youth Risk Behavior Web-based Survey were compared regarding their histories of subjective stress levels, sadness or despair, suicidal thoughts, suicide planning, and suicide attempts.

### Survey

#### Exposure

In 2019 and 2020, youth participants were selected as previously described to represent the entire youth population in Korea. The 2019 participants were not followed up. The 2020 participants were newly selected from the entire Korean youth population. The KYRBWS of 2019 was conducted from June 3 through July 12. KYRBWS data from 2020 were collected from August 3 through November 13.^18^

#### Outcomes

The participants were asked about their subjective stress, which they rated according to the following levels: very severe, severe, moderate, little, or no stress. They were asked if they had felt sadness or despair that was sufficient to make them pause their social lives for a period of 2 weeks within the past 12 months. In addition, the participants were asked if they had considered suicide seriously within the past 12 months, if they had planned suicide in detail within the past 12 months, and if they had attempted suicide within the past 12 months.

#### Covariates

Body mass index (BMI) was calculated as weight in kilograms divided by height in meters squared. Days of physical activity were measured as the number of days over the past 7 days that the participants had exercised for more than 60 minutes at an intensity high enough to increase their heart rate or respiration. Both the mean sedentary time (hours/day) for study and the mean sedentary time for leisure were calculated as 5/7 of the time spent on weekdays plus 2/7 of the time spent on weekends.^19^ Sleep time was calculated as 5/7 of the time spent on weekdays plus 2/7 of the time spent on weekends.^19^ The self-reported economic level was measured at 3 levels: high, middle, and low. The educational levels of the father and mother were categorized into 3 groups: unknown, missing, or below middle school; high school or college; and more than college. Scholastic performance was divided into 5 groups from highest to lowest. Subjective self-reported heath status was categorized into 4 levels, ranging from very healthy to unhealthy.

### Statistical Analysis

The general characteristics of the 2019 and 2020 participants were compared using linear regression analysis with complex sampling to represent the entire population, as this study was designed to use weighted values with stratified, clustered, or multistage sampling methods.^20^ The χ^2^ test with Rao-Scott correction was also used for the same reason.

The ORs for subjective stress, sadness or despair, suicidal thoughts, suicide planning, and suicide attempts of the 2020 participants were compared with those of the 2019 participants using multiple logistic regression analysis with complex sampling using the weighted value of each participant.^21^ Crude and adjusted models (for age, BMI, physical exercise, sedentary time for study and leisure, sleep time, sex, economic level, educational levels of the father and mother, scholastic performance, and subjective health status) were designed. Subgroup analyses by sex, economic level, and scholastic performance were designed.

Two-tailed analyses were conducted, and *P* < .05 was considered to indicate significance; 95% CIs were also calculated. The weights recommended by the KYRBWS were applied; thus, all results are presented as weighted values. The data were analyzed using SPSS version 25.0 (IBM) from January to February 2021.

## Results

A total of 92 659 Korean youth participants were included in this study; 48 443 youths in the 2019 KYRBWS (24 917 male youths [51.3%]; mean [SD] age, 15.0 [1.7] years) were compared with 44 216 youths in the 2020 KYRBWS (23 103 male youths [52.5%]; mean [SD] age, 15.1 [1.7] years). The stress levels were different between the 2019 and 2020 participants (Table 1). The severe and very severe stress levels were higher in the 2019 participants than in the 2020 participants (28.3% [n = 13 605] vs 25.7% [n = 11 231] for severe stress and 11.1% [n = 5364] vs 7.5% [n = 3353] for very severe stress; *P* < .001). Sadness or despair, suicidal thoughts, suicide planning, and suicide attempts were higher in the 2019 participants than in the 2020 participants. A total of 27.5% of the 2019 participants (n = 13 232) and 23.9% of the 2020 participants (n = 10 609) experienced sadness or despair (*P* < .001). A total of 12.6% of the 2019 participants (n = 6092) and 10.1% of the 2020 participants (n = 4517) reported a history of suicidal thoughts (*P* < .001). A total of 3.5% of the 2019 participants (n = 1721) and 3.1% of the 2020 participants (n = 1396) reported a history of suicide planning (*P* = .002). A total of 2.6% of the 2019 participants (n = 1283 ) and 1.7% of the 2020 participants (n = 760) reported a history of suicide attempts (*P* < .001).

**Table 1.** General Characteristics of Participants

General characteristics^a^	Participants, No. (%)		*P* value
	2019	2020	
Total No.	48 443 (100.0)	44 216 (100.0)	
Age, mean (SD), y	15.0 (1.7)	15.1 (1.7)	<.001^b^
BMI, mean (SD)	21.4 (3.5)	21.6 (3.7)	<.001^b^
Physical exercise, mean (SD), d/wk	2.0 (2.1)	1.9 (2.1)	<.001^b^
Sedentary time, mean (SD), h/d			
For study	6.6 (3.7)	6.0 (3.3)	<.001^b^
For leisure	3.3 (2.2)	4.2 (2.7)	<.001^b^
Sleep time, mean (SD), h/d	7.0 (1.5)	6.9 (1.5)	<.001^b^
Sex			
Male	24 917 (51.3)	23 103 (52.5)	.50
Female	23 526 (48.7)	21 113 (47.5)	
Economic level			
High	18 992 (39.6)	17 381 (40.3)	.32
Middle	23 376 (48.1)	21 228 (47.5)	
Low	6075 (12.3)	5607 (12.2)	
Educational level of father			
Unknown, missing, below middle school	23 775 (48.3)	17 957 (39.7)	<.001^c^
High school	7385 (14.8)	7519 (16.2)	
College or greater	17 283 (36.8)	18 740 (44.0)	
Educational level of mother			
Unknown, missing, below middle school	23 050 (47.0)	16 925 (37.5)	<.001^c^
High school	8637 (17.7)	8941 (19.6)	
College or greater	16 756 (35.3)	18 350 (42.9)	
Scholastic performance			
Highest	6464 (13.1)	5625 (12.7)	.12
Middle high	12 407 (25.5)	11 157 (25.5)	
Middle	14 697 (30.4)	13 496 (30.4)	
Middle low	10 519 (21.8)	9995 (22.5)	
Lowest	4356 (9.1)	3943 (8.9)	
Subjective health status			
Very healthy	12 911 (26.4)	12 283 (27.3)	.07
Healthy	21 444 (44.2)	19 131 (43.4)	
Normal	10 749 (22.4)	9742 (22.2)	
Unhealthy	3339 (70.)	3060 (7.1)	
Stress level			
No stress	1805 (3.6)	1597 (3.5)	<.001^c^
Little	7579 (15.4)	8128 (18.2)	
A little	20 090 (41.6)	19 907 (45.2)	
Severe	13 605 (28.3)	11 231 (25.7)	
Very severe	5364 (11.1)	3353 (7.5)	
Sadness or despair	13 232 (27.5)	10 609 (23.9)	<.001^c^
Thinking of suicide	6092 (12.6)	4517 (10.1)	<.001^c^
Plan of suicide	1721 (3.5)	1396 (3.1)	.002^c^
Suicide attempt	1283 (2.6)	760 (1.7)	<.001^c^

Abbreviation: BMI, body mass index (calculated as weight in kilograms divided by height in meters squared).

^a^ Estimated mean or prevalence adjusted recommended weighted value.

^b^ Linear regression analysis with complex sampling.

^c^ χ^2^ test with Rao-Scott correction.

The 2020 participants had engaged in fewer mean (SD) days of exercise than the 2019 participants (1.9 [2.1] days/week vs 2.0 [2.1] days/week; *P* < .001). Mean (SD) sedentary time for study was shorter in the 2020 participants than in the 2019 participants, whereas mean (SD) sedentary time for leisure was longer in the 2020 participants than in the 2019 participants (study: 6.0 [3.3] hours/day in 2020 vs 6.6 [3.7] hours/day in 2019; *P* < .001; leisure: 4.2 [2.7] hours/day in 2020 vs 3.3 [2.2] hours/day in 2019; *P* < .001). Mean (SD) sleep time was shorter in the 2020 participants than in the 2019 participants (6.9 [1.5] hours/day vs 7.0 [1.5] hours/day; *P* < .001). Mean (SD) BMI was higher in the 2020 participants than in the 2019 participants (21.6 [3.7] vs 21.4 [3.5]; *P* < .001). There was no difference in scholastic performance or subjective health status between the 2019 and 2020 participants.

The odds of experiencing little and moderate degrees of subjective stress were 1.20 (95% CI, 1.11-1.30) and 1.09 (95% CI, 1.02-1.18) times higher, respectively, in 2020 than in 2019 (*P* < .001). However, the odds of experiencing severe and very severe degrees of subjective stress were 0.90 (95% CI, 0.83-0.97) and 0.65 (95% CI, 0.60-0.72) times lower, respectively, in 2020 than in 2019 (*P* < .001) (Table 2). The odds of experiencing sadness or despair was 0.81 (95% CI, 0.78-0.84) times lower in 2020 than in 2019 (*P* < .001). The odds of suicidal thoughts, suicide planning, and suicide attempts were lower in 2020 than in 2019 (suicidal thoughts: adjusted OR [aOR], 0.77 [95% CI, 0.73-0.80];*P* < .001; suicide planning: aOR, 0.88 [95% CI, 0.81-0.96]; *P* < .001; suicide attempts: aOR, 0.64 [95% CI, 0.58-0.70]; *P* = .001).

**Table 2.** Odds of Stress Level, Sadness or Despair, Thinking of Suicide, Plan of Suicide, and Suicide Attempt for 2019 vs 2020 Participants

Variables	Event/total (%)		Crude OR (95% CI)	*P* value^a^	Adjusted OR (95% CI)^b^	*P* value^a^
	2019	2020				
Subjective stress						
None	NA	NA	1 [Reference]	<.001	1 [Reference]	<.001
Little	7579/48 443 (15.6)	8128/44 216 (18.4)	1.24 (1.14-1.34)		1.20 (1.11-1.30)	
A little	20 090/48 443 (41.5)	19 907/44 216 (45.0)	1.14 (1.06-1.24)		1.09 (1.02-1.18)	
Severe	13 605/48 443 (28.1)	11 231/44 216 (25.4)	0.95 (0.87-1.04)		0.90 (0.83-0.97)	
Very severe	5364/48 443 (11.1)	3353/44 216 (7.6)	0.71 (0.65-0.78)		0.65 (0.60-0.72)	
Sadness or despair	13 232/48 443 (27.3)	10 609/44 216 (24.0)	0.83 (0.80-0.86)	<.001	0.81 (0.78-0.84)	<.001
Thinking of suicide	6092/48 443 (12.6)	4517/44 216 (10.2)	0.78 (0.74-0.82)	<.001	0.77 (0.73-0.80)	<.001
Plan of suicide	1721/48 443 (3.6)	1396/44 216 (3.2)	0.89 (0.82-0.96)	.002	0.88 (0.81-0.96)	.001
Suicide attempt	1283/48 443 (2.6)	760/44 216 (1.7)	0.65 (0.59-0.71)	<.001	0.64 (0.58-0.70)	<.001

Abbreviations: NA, not applicable; OR, odds ratio.

^a^ Calculated using multiple logistic regression analysis with complex sampling; the threshold for statistical significance was *P* < .05.

^b^ Adjusted for age, body mass index, physical exercise, sedentary time for study and leisure, sleep time, sex, economic level, educational level of father and mother, scholastic performance, and subjective health status.

Both the male and female groups showed lower odds of experiencing severe stress, sadness or despair, and suicidality in 2020 than in 2019 (Table 3). In the male group, the 2020 participants had lower odds of very severe stress (aOR, 0.68 [95% CI, 0.61-0.77], *P* < .001 ), sadness or despair (aOR, 0.84 [95% CI, 0.80-0.89], *P* < .001), suicidal thoughts (aOR, 0.79 [95% CI, 0.73-0.85], *P* < .001), and suicide attempts (aOR, 0.67 [95% CI, 0.56-0.79], *P* < .001) than the 2019 participants. In the female group, the 2020 participants showed lower odds of severe stress (aOR, 0.77 [95% CI, 0.66-0.89], *P* < .001), very severe stress (aOR, 0.58 [95% CI, 0.49-0.69], *P* < .001 ), sadness or despair (aOR, 0.79 [95% CI, 0.75-0.83], *P* < .001), suicidal thoughts (aOR, 0.75 [95% CI, 0.71-0.80], *P* < .001), suicide planning (aOR, 0.86 [95% CI, 0.77-0.95]; *P* = .005), and suicide attempts (aOR, 0.62 [95% CI, 0.55-0.70]; *P* < .001) than the 2019 participants. Regarding income levels, all groups (ie, the high-, middle-, and low-income groups) showed lower odds of severe stress, sadness or despair, suicidal thoughts, and suicide attempts in the 2020 participants than in the 2019 participants (Table 4). In addition, according to scholastic performance, all groups (ie, the highest-, middle high-, middle, middle low-, and low-scoring groups, demonstrated lower odds of severe stress, sadness or despair, suicidal thoughts, and suicide attempts in the 2020 participants than in the 2019 participants (eTable in the Supplement).

**Table 3.** Odds of Stress Level, Sadness or Despair, Thinking of Suicide, Plan of Suicide, and Suicide Attempt for 2019 vs 2020 Participants by Sex

Variables	Event/total (%)		Crude OR (95% CI)	*P* value^a^	Adjusted OR (95% CI)^b^	*P* value^a^
	2019	2020				
Men (n = 48 020)						
Subjective stress						
None	NA	NA	1 [Reference]	<.001	1 [Reference]	<.001
Little	4947/24 917 (19.9)	5231/23 103 (22.6)	1.30 (1.19-1.42)		1.26 (1.15-1.38)	
A little	10 874/24 917 (43.6)	10 453/23 103 (45.2)	1.18 (1.08-1.28)		1.11 (1.02-1.21)	
Severe	5622/24 917 (22.6)	4933/23 103 (21.4)	1.08 (0.98-1.19)		1.00 (0.91-1.10)	
Very severe	2053/24 917 (8.2)	1290/23 103 (5.6)	0.77 (0.69-0.86)		0.68 (0.61-0.77)	
Sadness or despair	5268/24 917 (21.1)	4389/23 103 (19.0)	0.86 (0.82-0.91)	<.001	0.84 (0.80-0.89)	<.001
Thinking of suicide	2171/24 917 (8.7)	1727/23 103 (7.5)	0.83 (0.77-0.90)	<.001	0.79 (0.73-0.85)	<.001
Plan of suicide	637/24 917 (2.6)	552/23 103 (2.4)	0.95 (0.84-1.07)	.38	0.92 (0.80-1.04)	.18
Suicide attempt	378/24 917 (1.5)	254/23 103 (1.1)	0.71 (0.60-0.83)	<.001	0.67 (0.56-0.79)	<.001
Women (n = 44 639)						
Subjective stress						
None	NA	NA	1 [Reference]	<.001	1 [Reference]	<.001
Little	2632/23 526 (11.2)	2897/21 113 (13.7)	1.05 (0.90-1.22)		1.06 (0.91-1.23)	
A little	9216/23 526 (39.2)	9454/21 113 (44.8)	0.99 (0.85-1.14)		1.00 (0.87-1.17)	
Severe	7983/23 526 (33.9)	6298/21 113 (29.8)	0.75 (0.65-0.88)		0.77 (0.66-0.89)	
Very severe	3311/23 526 (14.1)	2063/21 113 (9.8)	0.59 (0.50-0.69)		0.58 (0.49-0.69)	
Sadness or despair	7964/23 526 (33.9)	6220/21 113 (29.5)	0.81 (0.77-0.85)	<.001	0.79 (0.75-0.83)	<.001
Thinking of suicide	3921/23 526 (16.7)	2790/21 113 (13.2)	0.76 (0.71-0.81)	<.001	0.75 (0.71-0.80)	<.001
Plan of suicide	1084/23 526 (4.6)	844/21 113 (4.0)	0.86 (0.77-0.95)	.003	0.86 (0.77-0.95)	.005
Suicide attempt	905/23 526 (3.8)	506/21 113 (2.4)	0.63 (0.56-0.70)	<.001	0.62 (0.55-0.70)	<.001

Abbreviation: OR, odds ratio.

^a^ Calculated using multiple logistic regression analysis with complex sampling; the threshold for statistical significance was *P* < .05.

^b^ Adjusted for age, body mass index, physical exercise, sedentary time for study and leisure, sleep time, sex, economic level, educational level of father and mother, scholastic performance, and subjective health status.

**Table 4.** Odds of Stress Level, Sadness or Despair, Thinking of Suicide, Plan of Suicide, and Suicide Attempt for 2019 vs 2020 Participants by Economic Level

Variables	Event/total (%)		Crude OR (95% CI)	*P* value^a^	Adjusted OR (95% CI)^b^	*P* value^a^
	2019	2020				
High income (n = 36 373)						
Subjective stress						
None	NA	NA	1 [Reference]	<.001	1 [Reference]	<.001
Little	3481/18 992 (18.3)	3618/17 381 (20.8)	1.22 (1.09-1.35)		1.18 (1.06-1.32)	
A little	7726/18 992 (40.7)	7731/17 381 (44.5)	1.17 (1.06-1.30)		1.10 (1.00-1.22)	
Severe	4926/18 992 (25.9)	4018/17 381 (23.1)	0.95 (0.84-1.06)		0.88 (0.79-0.98)	
Very severe	1858/18 992 (9.8)	1141/17 381 (6.6)	0.72 (0.63-0.82)		0.65 (0.57-0.74)	
Sadness or despair	4885/18 992 (25.7)	3841/17 381 (22.1)	0.82 (0.78-0.86)	<.001	0.79 (0.75-0.83)	<.001
Thinking of suicide	2054/18 992 (10.8)	1533/17 381 (8.8)	0.79 (0.74-0.86)	<.001	0.77 (0.71-0.83)	<.001
Plan of suicide	586/18 992 (3.1)	472/17 381 (2.7)	0.92 (0.81-1.04)	.20	0.88 (0.77-1.01)	.07
Trial of suicide	418/18 992 (2.2)	262/17 381 (1.5)	0.67 (0.57-0.79)	<.001	0.62 (0.53-0.74)	<.001
Middle income (n = 44 604)						
Subjective stress						
None	NA	NA	1 [Reference]	<.001	1 [Reference]	<.001
Little	3481/23 376 (14.9)	3818/21 228 (18.0)	1.23 (1.09-1.38)		1.19 (1.05-1.34)	
A little	10 196/23 376 (43.6)	9928/21 228 (46.8)	1.09 (0.97-1.22)		1.05 (0.94-1.18)	
Severe	6607/23 376 (28.3)	5373/21 228 (25.3)	0.92 (0.81-1.04)		0.88 (0.79-0.99)	
Very severe	2410/23 376 (10.3)	1492/21 228 (7.0)	0.69 (0.60-0.79)		0.64 (0.56-0.73)	
Sadness or despair	6042/23 376 (25.8)	4888/21 228 (23.0)	0.85 (0.80-0.89)	<.001	0.83 (0.79-0.87)	<.001
Thinking of suicide	2732/23 376 (11.7)	1995/21 228 (9.4)	0.77 (0.72-0.83)	<.001	0.75 (0.70-0.81)	<.001
Plan of suicide	724/23 376 (3.1)	596/21 228 (2.8)	0.88 (0.79-0.99)	.04	0.89 (0.79-1.00)	.05
Suicide attempt	521/23 376 (2.2)	314/21 228 (1.5)	0.66 (0.57-0.77)	<.001	0.66 (0.57-0.77)	<.001
Low income (n = 11 682)						
Subjective stress						
None	NA	NA	[Reference]	<.001	[Reference]	<.001
Little	617/6075 (10.2)	692/5607 (12.3)	1.44 (1.07-1.95)		1.43 (1.06-1.94)	
A little	2168/6075 (35.7)	2248/5607 (40.1)	1.33 (1.00-1.76)		1.32 (0.99-1.77)	
Severe	2072/6075 (34.1)	1840/5607 (32.8)	1.13 (0.85-1.52)		1.14 (0.85-1.53)	
Very severe	1096/6075 (18.0)	720/5607 (12.8)	0.79 (0.59-1.07)		0.79 (0.59-1.07)	
Sadness or despair	2305/6075 (37.9)	1880/5607 (33.5)	0.80 (0.74-0.86)	<.001	0.81 (0.75-0.88)	<.001
Thinking of suicide	1306/6075 (21.5)	989/5607 (17.6)	0.78 (0.70-0.86)	<.001	0.79 (0.71-0.87)	<.001
Plan of suicide	411/6075 (6.8)	328/5607 (5.8)	0.84 (0.72-0.98)	.02	0.84 (0.72-0.99)	.04
Suicide attempt	344/6075 (5.7)	184/5607 (3.3)	0.58 (0.49-0.70)	<.001	0.60 (0.50-0.72)	<.001

Abbreviations: NA, not applicable; OR, odds ratio.

^a^ Calculated using multiple logistic regression analysis with complex sampling; the threshold for statistical significance was *P* < .05.

^b^ Adjusted for age, body mass index, physical exercise, sedentary time for study and leisure, sleep time, sex, economic level, educational level of father and mother, scholastic performance, and subjective health status.

## Discussion

During the early COVID-19 pandemic period, the odds of severe stress, sadness or despair, and suicidality were lower than those during the pre-COVID-19 period in Korean youths. Although mild stress (little or moderate stress) increased, high levels of stress (severe and very severe stress) decreased during the COVID-19 pandemic period compared with the stress levels during the pre-COVID-19 pandemic period. The lower odds of severe stress, sadness or despair, and suicidality during the COVID-19 pandemic period were consistent in all subgroups by sex, income, and scholastic performance. Multiple factors may have induced lower severe stress and suicidality during the COVID-19 pandemic period in youths.

Social distancing policies and school closures to restrain the spread of COVID-19 may have decreased the severe stress from social activities and academic burdens in youths. In our study, physical exercise decreased during the COVID-19 pandemic period (2.0 days/week in 2019 vs 1.9 days/week in 2020). However, sedentary time for leisure increased and sedentary time for study decreased during the COVID-19 pandemic period. Increased leisure time and decreased study time could decrease stress from academic burdens in youths. The Korean government mandated complete school closures from March 2, 2020, until May 13, 2020, and both offline and online classes with partial school attendance were maintained (as of May 2021).^22^ School closures can alleviate stress and suicidality in youths by reducing academic burdens and school bullying.^4,12^ Academic stress was reported to be a major risk factor for suicide in Korean youths.^23^ During the COVID-19 pandemic, the quality of peer relationships was no longer associated with life satisfaction in school-aged children.^12^ In addition, increased family coherence due to spending more time with family could attenuate stress and suicidality in youths during school closures.^16^ During school closures, approximately 21.4% of school-aged children were found to be more satisfied with life and parent-children discussions.^16^ In addition, awareness of home quarantine was positively correlated with life satisfaction but negatively correlated with psychopathologic symptoms.^16^

Health-protective behavior, such as the use of face masks, hand hygiene, and nutritional intake, can increase psychological well-being. Adequate knowledge of COVID-19 and proper use of face masks were identified as protective factors against depression during the COVID-19 outbreak.^24^ Due to the lockdowns of schools and private educational institutes, youths may have been less exposed to sleep deprivation and irregular diets. These factors could have reduced stress and suicidality in youths. Sleep quality was related to stress and dietary behaviors during the COVID-19 pandemic.^25^ In addition, the pulling-together effect or honeymoon phase during the early pandemic period may have impacted the lower stress and suicidality observed in this study.^4,8,26^ Several epidemiologic studies demonstrated unchanged or decreased suicide rates or suicidality during the early months of the COVID-19 pandemic.^4,8^ The psychiatric response to disasters has been divided into 4 phases: the heroic phase, honeymoon phase, disillusionment phase, and restoration phase.^27^ The honeymoon phase refers to a phase characterized by hopefulness and optimism from a spirit of togetherness.^28^ This hopefulness and optimism were suggested to be related to faith in and relief from rescue management of the community and sympathy for administrators to cope with disasters.^27^ Such community ties could be generated from common experiences and cooperation to cope with the disaster.^26^ The 2020 KYRBWS was conducted during the first and second waves of the COVID-19 pandemic, from August 3, 2020, to November 13, 2020.^18^ Thus, national actions to suppress SARS-CoV-2 infection, financial support, and public campaigns may have encouraged social cohesion during the early phase of the COVID-19 pandemic.

### Strengths and Limitations

This study had some strengths and limitations. It was based on a large, representative youth participant cohort. Many covariates, including age, sex, BMI, physical exercise, sedentary time for study and leisure, sleep time, economic level, educational levels of the father and mother, scholastic performance, and subjective health status, were considered to attenuate the possible confounding effects. However, the survey was based on a self-reported questionnaire. Subjective stress level was measured using a simple question and was not based on a scientific scale. Although samples representative of the Korean youth population were selected in both years, the study participant was cross-sectional, and a longitudinal follow-up survey could not be conducted for each person. In addition, the survey was conducted within a year after the COVID-19 pandemic period, but the long-term effects of the COVID-19 pandemic on the stress and suicidality of youths warrant a longer follow-up study. Finally, the present study population consisted of Korean youths of a single ethnicity. Because the number of patients with COVID-19 in Korea has been controlled within 1300 persons per day as of May 2021, the disease burden of COVID-19 could be lower than that of other countries with higher infection rates.^29,30^ In addition, socioeconomic and cultural aspects could also have influenced the impact of the COVID-19 pandemic on stress and suicidality.

## Conclusions

In this cross-sectional study, severe stress and suicidality were lower in Korean youths during the early COVID-19 pandemic period than before the pandemic. Reduced academic and social burdens and enhanced family coherence and health-seeking behavior could have mediated the alleviation of severe stress and suicidality in youths. The potential long-term effects of the COVID-19 pandemic warrant additional follow-up studies.

## Notes

Supplement. **eAppendix.** Korea Youth Risk Behavior Web-based Survey
eReferences
**eTable.** Odds Ratios of Stress Level, Sadness or Despair, Thoughts of Suicide, Plan of Suicide, and Trial of Suicide in 2020 Compared to 2019 in Subgroup Analyses by Scholastic Performance
