# Supplementary material for: Comparison of Stress and Suicide-Related Behaviors Among Korean Youths Before and During the COVID-19 Pandemic
Source: JAMA Netw Open. 2021 Dec 13;4(12):e2136137. doi: 10.1001/jamanetworkopen.2021.36137 (PMC8669543; doi:10.1001/jamanetworkopen.2021.36137)
Supplement: Supplement. — eAppendix. Korea Youth Risk Behavior Web-based Survey eReferences eTable. Odds Ratios of Stress Level, Sadness or Despair, Thoughts of Suicide, Plan of Suicide, and Trial of Suicide in 2020 Compared to 2019 in Subgroup Analyses by Scholastic Performance [file jamanetwopen-e2136137-s001.pdf]

Supplemental Online Content

Kim SY, Kim HR, Park B, Choi HG. Comparison of stress and suicide-related behaviors among Korean youths before and during the COVID-19 pandemic. *JAMA Netw Open.* 2021;4(12):e2136137. doi:10.1001/jamanetworkopen.2021.36137

eAppendix. Korea Youth Risk Behavior Web-based Survey

eReferences

eTable. Odds Ratios of Stress Level, Sadness or Despair, Thoughts of Suicide, Plan of Suicide, and Trial of Suicide in 2020 Compared to 2019 in Subgroup Analyses by Scholastic Performance

This supplemental material has been provided by the authors to give readers additional information about their work.

© 2021 Kim SY et al. *JAMA Network Open.*

eAppendix. Korea Youth Risk Behavior Web-based Survey

All Korea Youth Risk Behavior Web-based Survey (KYRBWS) data analyses were conducted in accordance with the guidelines and regulations provided by the The Institutional Review Board of the Centers for Disease Control and Prevention of Korea (KCDC). The understanding, reliability and validity of each question were investigated by the KCDC to verify the applicability of the surveys[1].

This cross-sectional study used data from KYRBWS and covered the nation using statistical methods based on designed sampling and adjusted weighted values. The KYRBWS obtains data from South Korean adolescents using stratified, two-stage (schools and classes) clustered sampling based on data from the Education Ministry. Sampling was weighted by statisticians, who performed post-stratification analyses and considered the non-response rates and extreme values. Data from the 2019, and 2020 KYRBWS were analyzed. Details of the sampling methods are described on the KYRBWS website[1]. The KCDC collected the data, and Korean adolescents from 7th through 12th grade completed the self-administered questionnaire voluntarily and anonymously. The validity and reliability of KYRBWS have been documented by other studies[2,3].

eReferences

1 Park, S. W. *Reliability and Validity of the Korea Youth Risk Behavior Web-based Survey Questionnaire 2009*, <http://yhs.cdc.go.kr/ > (2009).

2 Bae, J. *et al.* Validity of self-reported height, weight, and body mass index of the Korea

© 2021 Kim SY et al. *JAMA Network Open.*

Youth Risk Behavior Web-based Survey questionnaire. *J Prev Med Public Health* **43**, 396-402, doi:10.3961/jpmph.2010.43.5.396 (2010).

3 Bae, J. *et al.* Test-retest reliability of a questionnaire for the Korea Youth Risk Behavior Web-based Survey. *J Prev Med Public Health* **43**, 403-410, doi:10.3961/jpmph.2010.43.5.403 (2010).

© 2021 Kim SY et al. *JAMA Network Open.*

eTable. Odd ratios of stress level, sadness or despair, thinking of suicide, plan of suicide, and trial of suicide of 2002 compared to 2019 in subgroup analyses by scholastic performance

Variables		Event/total (n, %)		OR (95% CI)			
		2019	2020	Crude	P-value	Adjusted [b]	P-value
Highest score (n = 12,089)							
Subjective stress (reference = no)					<.001 [a]		<.001 [a]
	Little	1,229/6,464 (19.0)	1,187/5,625 (21.1)	1.23 (1.04-1.45)		1.15 (0.97-1.36)	
	A little	2,533/6,464 (39.2)	2,397/5,625 (42.6)	1.22 (1.04-1.44)		1.13 (0.96-1.34)	
	Severe	1,606/6,464 (24.8)	1,316/5,625 (23.4)	1.06 (0.90-1.26)		0.96 (0.80-1.15)	
	Very severe	667/6,464 (10.3)	388/5,625 (6.9)	0.74 (0.61-0.92)		0.67 (0.54-0.83)	
Sadness or despair		1,427/6,464 (22.1)	1,133/5,625 (20.1)	0.88 (0.80-0.96)	.005 [a]	0.85 (0.78-0.94)	.001 [a]
Thinking of suicide		677/6,464 (10.5)	514/5,625 (9.1)	0.87 (0.77-0.99)	.04 [a]	0.84 (0.74-0.96)	.009 [a]
Plan of suicide		202/6,464 (3.1)	156/5,625 (2.8)	0.92 (0.74-1.15)	.48	0.91 (0.72-1.15)	.43
Trial of suicide		134/6,464 (2.1)	77/5,625 (1.4)	0.63 (0.47-0.84)	.002 [a]	0.61 (0.46-0.83)	.002 [a]
Middle high score (n = 23,564)							
Subjective stress (reference = no)					<.001 [a]		<.001 [a]
	Little	1,999/12,407 (16.1)	2,239/11,157 (20.1)	1.28 (1.10-1.49)		1.26 (1.08-1.47)	

© 2021 Kim SY et al. *JAMA Network Open.*

A little	5,354/12,407 (43.2)	5,159/11,157 (46.2)	1.08 (0.94-1.26)		1.06 (0.92-1.22)	
Severe	3,454/12,407 (27.8)	2,686/11,157 (24.1)	0.87 (0.75-1.01)		0.84 (0.73-0.98)	
Very severe	1,161/12,407 (9.4)	686/11,157 (6.1)	0.65 (0.55-0.77)		0.63 (0.53-0.75)	
Sadness or despair	3,086/12,407 (24.9)	2,381/11,157 (21.3)	0.81 (0.76-0.87)	<.001 [a]	0.79 (0.74-0.85)	<.001 [a]
Thinking of suicide	1,354/12,407 (10.9)	1,012/11,157 (9.1)	0.80 (0.73-0.87)	<.001 [a]	0.80 (0.73-0.88)	<.001 [a]
Plan of suicide	367/12,407 (3.0)	269/11,157 (2.4)	0.78 (0.66-0.92)	<.001 [a]	0.79 (0.66-0.94)	.007 [a]
Trial of suicide	233/12,407 (1.9)	149/11,157 (1.3)	0.67 (0.54-0.84)	<.001 [a]	0.66 (0.52-0.83)	<.001 [a]
Middle score (n = 28,193)						
Subjective stress (reference = no)				<.001 [a]		<.001 [a]
Little	2,338/14,697 (15.9)	2,440/13,496 (18.1)	1.13 (0.98-1.30)		1.12 (0.97-1.29)	
A little	6,397/14,697 (43.5)	6,361/13,496 (47.1)	1.10 (0.95-1.26)		1.06 (0.93-1.22)	
Severe	4,011/14,697 (27.3)	3,384/13,496 (25.1)	0.92 (0.80-1.07)		0.90 (0.78-1.03)	
Very severe	1,477/14,697 (10.0)	839/13,496 (6.2)	0.63 (0.53-0.74)		0.60 (0.51-0.70)	
Sadness or despair	3,801/14,697 (25.9)	3,035/13,496 (22.5)	0.83 (0.79-0.87)	<.001 [a]	0.83 (0.78-0.89)	<.001 [a]
Thinking of suicide	1,692/14,697 (11.5)	1,185/13,496 (8.8)	0.73 (0.67-0.79)	<.001 [a]	0.72 (0.66-0.79)	<.001 [a]
Plan of suicide	454/14,697 (3.1)	371/13,496 (2.7)	0.90 (0.78-1.04)	.14	0.89 (0.77-1.04)	.13
Trial of suicide	354/14,697 (2.4)	194/13,496 (1.4)	0.60 (0.50-0.72)	<.001 [a]	0.59 (0.49-0.71)	<.001 [a]

© 2021 Kim SY et al. *JAMA Network Open.*

Middle low score (n = 20,514)						
Subjective stress (reference = no)				<.001 [a]		<.001 [a]
Little	1,441/10,519 (13.7)	1,636/9,995 (16.4)	1.32 (1.10-1.59)		1.31 (1.08-1.58)	
A little	4,306/10,519 (40.9)	4,490/9,995 (44.9)	1.20 (1.00-1.43)		1.18 (0.99-1.42)	
Severe	3,217/10,519 (30.6)	2,739/9,995 (27.4)	0.99 (0.82-1.19)		0.96 (0.79-1.15)	
Very severe	1,245/10,519 (11.8)	875/9,995 (8.8)	0.81 (0.66-1.00)		0.76 (0.62-0.94)	
Sadness or despair	3,281/10,519 (31.2)	2,744/9,995 (27.5)	0.81 (0.76-0.87)	<.001 [a]	0.79 (0.74-0.85)	<.001 [a]
Thinking of suicide	1,544/10,519 (14.7)	1,177/9,995 (11.8)	0.78 (0.72-0.86)	<.001 [a]	0.76 (0.69-0.83)	<.001 [a]
Plan of suicide	432/10,519 (4.1)	372/9,995 (3.7)	0.95 (0.82-1.09)	.45	0.93 (0.81-1.07)	.32
Trial of suicide	335/10,519 (3.2)	203/9,995 (2.0)	0.67 (0.57-0.80)	<.001 [a]	0.67 (0.56-0.80)	<.001 [a]
Low score (n = 8,299)						
Subjective stress (reference = no)				<.001 [a]		<.001 [a]
Little	572/4,356 (13.1)	626/3,943 (15.9)	1.19 (0.92-1.54)		1.24 (0.95-1.61)	
A little	1,500/4,356 (34.4)	1,500/3,943 (38.0)	1.08 (0.85-1.39)		1.13 (0.88-1.46)	
Severe	1,317/4,356 (30.2)	1,106/3,943 (28.0)	0.91 (0.71-1.18)		0.95 (0.73-1.24)	
Very severe	814/4,356 (18.7)	565/3,943 (14.3)	0.70 (0.53-0.91)		0.70 (0.53-0.92)	
Sadness or despair	1,637/4,356 (37.6)	1,316/3,943 (33.4)	0.81 (0.73-0.89)	<.001 [a]	0.82 (0.74-0.90)	<.001 [a]

© 2021 Kim SY et al. *JAMA Network Open.*

Thinking of suicide	825/4,356 (18.9)	629/3,943 (16.0)	0.78 (0.69-0.88)	<.001 [a]	0.78 (0.69-0.88)	<.001 [a]
Plan of suicide	266/4,356 (6.1)	228/3,943 (5.8)	0.87 (0.73-1.05)	.15	0.90 (0.74-1.09)	.27
Trial of suicide	227/4,356 (5.2)	137/3,943 (3.5)	0.65 (0.52-0.80)	<.001 [a]	0.66 (0.53-0.82)	<.001 [a]

[a] Multiple logistic regression analysis with complex sampling, Significance at $P < 0.05$

[b] Adjusted for age, BMI, physical exercise, sedentary time for study and leisure, sleep time, sex, economic level, educational level of father and mother, scholastic performance, and subjective health status

© 2021 Kim SY et al. *JAMA Network Open.*
